# Clinical Features and Symptom Burden in Vietnamese Patients with Diarrhea-Predominant Irritable Bowel Syndrome: A Single-Center Cross-Sectional Study Using IBS-SSS and IBS-QoL Scores

**DOI:** 10.3390/jcm15082910

**Published:** 2026-04-11

**Authors:** Qui Huu Nguyen, Huong Tu Lam, Thuy Thi Thanh Trinh, Thong Duy Vo

**Affiliations:** 1 Department of Gastroenterology, University Medical Center Ho Chi Minh City, Ho Chi Minh City 72714, Vietnam; qui.nh@umc.edu.vn (Q.H.N.); thuy.ttt04@umc.edu.vn (T.T.T.T.); 2 Department of Internal Medicine, School of Medicine, University of Medicine and Pharmacy at Ho Chi Minh City, Ho Chi Minh City 72714, Vietnam; lamtuhuong@ump.edu.vn; 3 Cho Ray Hospital, Ho Chi Minh City 72714, Vietnam

**Keywords:** irritable bowel syndrome, quality of life, IBS-D, IBS-SSS, IBS-QoL

## Abstract

**Background/Objectives**. Diarrhea-predominant irritable bowel syndrome (IBS-D) significantly affects patients’ quality of life (QoL). However, data on disease severity and its correlation with QoL among Vietnamese patients remain limited. This study aimed to investigate the clinical characteristics, symptom severity, and the relationship between symptom burden and quality of life in patients with IBS-D in Vietnam. **Methods**. A cross-sectional study was conducted on patients diagnosed with IBS-D based on the Rome IV criteria at an outpatient clinic of a tertiary hospital. Disease severity and QoL were assessed using the IBS Symptom Severity Score (IBS-SSS) and the standardized Vietnamese version of the IBS Quality of Life (IBS-QoL) questionnaire, respectively. Clinical characteristics, comorbidities, and overlap syndromes were also recorded. **Results**. Among the 123 patients enrolled (mean age 42.6 ± 14.5 years; 55.3% female), the median IBS-SSS score was 175 (interquartile range: 140–225), and the median IBS-QoL score was 72 (interquartile range: 54–85). The prevalence of overlap syndromes was relatively high, with functional dyspepsia accounting for 46.3% and gastroesophageal reflux disease for 8.9%. A moderate inverse correlation was observed between IBS-SSS and IBS-QoL scores (r = −0.494; *p* < 0.001). Notably, patients with severe IBS (IBS-SSS ≥ 300) had significantly higher rates of smoking (44.4% vs. 13.2%; *p* = 0.012) and diabetes (22.2% vs. 5.3%; *p* = 0.047) compared to the non-severe group. **Conclusions**. IBS-D imposes a substantial symptom burden and significantly reduces the quality of life in Vietnamese patients, particularly among those with severe disease. The high prevalence of overlap syndromes, along with contributing factors like smoking and diabetes, further increase the complexity and severity of the condition.

## 1. Introduction

Irritable bowel syndrome (IBS) is defined as a disorder of gut–brain interaction, characterized by recurrent abdominal pain associated with defecation or changes in bowel habits [1,2,3]. It is one of the most common gastrointestinal disorders worldwide, with an estimated prevalence of about 4.1–10.1% of the population [2]. Although IBS does not increase mortality, the burden it imposes on quality of life (QoL) and healthcare costs is substantial, and has been reported to be comparable to that of other chronic organic diseases [2,4]. The global epidemiology of IBS shows considerable geographic variability, with prevalence estimates ranging from approximately 4% to 10% depending on diagnostic criteria and population characteristics. Recent studies based on Rome IV criteria suggest a lower prevalence compared to earlier definitions, reflecting stricter diagnostic thresholds. In Asia, IBS prevalence remains comparable but is often under-recognized due to differences in healthcare-seeking behavior.

In Vietnam, IBS represents a significant but under-characterized condition, with reported prevalence ranging from 7.2% to 17.4%. The diarrhea-predominant subtype (IBS-D) accounts for a substantial proportion of cases. Despite this burden, data on symptom severity and quality-of-life impairment remain limited.

Diarrhea-predominant irritable bowel syndrome (IBS-D) is one of the most common subtypes of IBS, accounting for approximately 30–40% of all IBS cases [2]. IBS-D is characterized by recurrent abdominal pain accompanied by changes in stool consistency (with loose or watery stools occurring in more than 25% of bowel movements) and increased stool frequency [3]. The disease burden of IBS-D is particularly severe due to the unpredictable nature of its symptoms. Studies have shown that patients with IBS-D are more profoundly affected socially and psychologically compared to other subtypes. The symptoms of urgency and fear of fecal incontinence often lead patients to avoid social activities, dining out, or traveling, resulting in isolation and a significant decline in QoL [2,5]. The clinical picture of IBS-D becomes even more complex due to the common presence of overlap syndromes and comorbid conditions [6]. There is a high rate of overlap between IBS and gastroesophageal reflux disease (GERD), functional dyspepsia (FD), as well as psychological disorders such as anxiety and depression. According to a study by Lackner et al., the interplay of these conditions plays a pivotal role in increasing the overall disease burden, sometimes to a degree even more severe than the gastrointestinal symptoms alone [7].

To objectively and comprehensively assess the disease burden of IBS, two specialized tools widely recommended in international guidelines are the IBS Symptom Severity Score (IBS-SSS) and the IBS Quality of Life questionnaire (IBS-QoL) [1,2]. IBS-SSS is a scoring system used to categorize disease severity (mild, moderate, or severe) based on the intensity of abdominal pain, bloating, and the overall impact on daily life. Patient stratification using the IBS-SSS plays a critical role in prognosis and in guiding appropriate treatment strategies [1,8]. In parallel, the IBS Quality of Life (IBS-QoL) questionnaire is a disease-specific tool designed to assess the impact of IBS across eight distinct domains, including health-related anxiety, dietary restrictions, and limitations in social activities. Studies have demonstrated a strong inverse correlation between the two scoring systems: higher IBS-SSS scores are consistently associated with lower IBS-QoL scores [7].

In Vietnam, IBS is a common healthcare concern, with an estimated prevalence ranging from 7.2% to 17.4%. Among these cases, the diarrhea-predominant subtype (IBS-D) accounts for approximately 30.3% to 67.1% [9,10]. Although international guidelines such as those from the AGA, BSG, and the Seoul Consensus have provided updated diagnostic recommendations, real-world data on the clinical characteristics and the correlation between IBS-SSS and IBS-QoL specifically in Vietnamese patients with IBS-D remain limited [11]. A clear understanding of the relationship between disease severity, quality of life impairment, and overlap syndromes serves as a crucial foundation for optimizing personalized treatment strategies.

Therefore, this study was conducted to investigate the clinical characteristics and classify disease severity using the IBS-SSS in patients with IBS-D in Vietnam, as well as to assess the relationship between IBS-SSS, IBS-QoL, and associated factors in this patient population.

## 2. Method

### 2.1. Study Design and Setting

A cross-sectional study was conducted on patients diagnosed with IBS-D who attended the Gastroenterology and Hepatology Outpatient Clinic at University Medical Center at Ho Chi Minh city from June 2025 to December 2025.

### 2.2. Study Population

Inclusion criteria were patients aged 18 years and older, diagnosed with IBS-D according to Rome IV criteria. Exclusion criteria included patients who did not consent to participate in the study; pregnant women; and patients with severe comorbidities or psychiatric disorders that impaired their ability to complete questionnaires.

### 2.3. Sample Size Calculation

The sample size was calculated using the formula for estimating a proportion in a population, with an expected prevalence of IBS-D of approximately 6% (based on an overall IBS prevalence of approximately 15% in the general population, with IBS-D accounting for around 40% of IBS cases) [9], a confidence level of 95%, and a margin of error of 5%. The minimum required sample size was calculated to be approximately 87 participants.

### 2.4. Criteria and Definitions

Patients were diagnosed with IBS-D according to the Rome IV criteria [3]:

- Recurrent abdominal pain, on average, at least 1 day per week in the last 3 months, associated with at least two of the following: related to defecation, associated with a change in stool frequency, or associated with a change in stool form. These criteria must be present during the past 3 months, with symptom onset at least 6 months before diagnosis;
- More than 25% of bowel movements classified as type 6 or 7 and less than 25% as type 1 or 2 on the Bristol Stool Form Scale.

The daily frequency of diarrhea was measured using the Bristol Stool Form Scale, with stool types 6 or 7 classified as episodes of diarrhea [1]. Abdominal pain severity was assessed using a 7-point Likert scale, where lower scores indicated greater pain intensity. Bloating severity was assessed using a 7-point Likert scale, with lower scores indicating more severe symptoms.

Quality of life was assessed using the validated Vietnamese version of the IBS-QoL questionnaire [12]. This instrument, originally developed by Drossman et al. [13], consists of 34 items covering 8 domains: dysphoria, interference with activity, body image, health worry, food avoidance, social reaction, sexual functioning, and relationships. Each domain is measured by a specific subset of the 34 items. Responses are rated according to the level of impact: none, mild, moderate, severe, or very severe. The total score from all items is transformed into a scale ranging from 0 to 100, with higher scores indicating better quality of life.

Disease severity was assessed using the IBS Symptom Severity Score (IBS-SSS), which evaluates five symptom domains over the past 10 days: severity and frequency of abdominal pain, bloating, dissatisfaction with bowel habits, and interference with daily life. Each item is rated on a visual analog scale ranging from 0 to 100. The total score ranges from 0 to 500 and is categorized into four levels: remission (<75), mild (75–174), moderate (175–299), and severe (≥300).

Comorbid conditions such as gastroesophageal reflux disease (GERD), functional dyspepsia (FD), *H. pylori* infection, anxiety disorders, and insomnia were identified based on physician diagnoses at the outpatient clinic.

### 2.5. Data Collection

**Figure 1:**
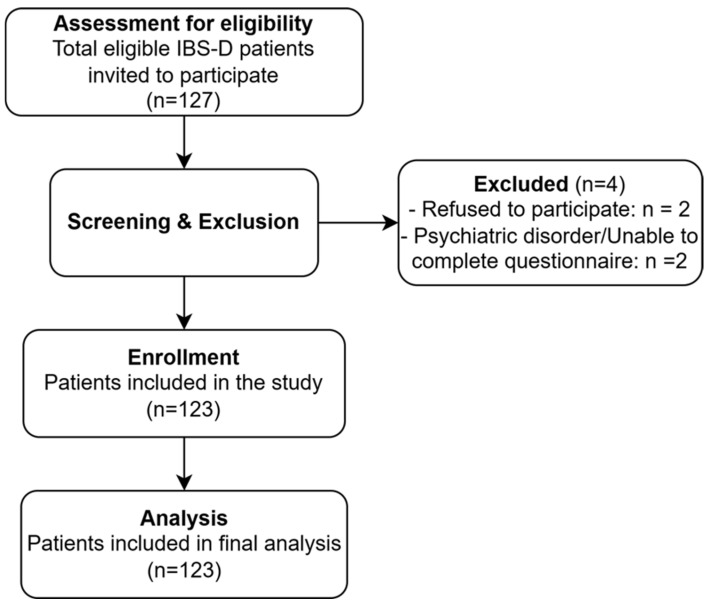
Flow chart of the study.

We collected data on patients, including age and sex, address, occupation, body mass index (BMI), signs and symptoms. Patients were interviewed to complete the IBS-SSS and IBS-QoL questionnaires. The flowchart of the study is demonstrated in Figure 1. Information regarding prior treatment was not systematically collected. Patients were managed according to routine clinical practice prior to enrollment, which may have included pharmacological therapies (e.g., antispasmodics, probiotics, antidiarrheal agents) and dietary modifications such as low-FODMAP or trigger-avoidance diets. However, treatment duration, specific regimens, and adherence were not recorded and were therefore not included in the analysis.

### 2.6. Ethics Statement

The study protocol adhered to the ethical principles outlined in the Declaration of Helsinki. The study was approved by the Ethics Committee for Biomedical Research of the University Medical Center at Ho Chi Minh city (Approval No.55/GCN-HDDD, signed on 25 April 2025).

### 2.7. Statistical Analysis

The study utilized Microsoft Excel for data entry, along with Stata 16.0 for analysis. Descriptive statistics were used for continuous and categorical variables, with comparisons conducted using appropriate tests such as Chi-square, Fisher’s exact test, *t*-test, or Mann–Whitney test. To examine the relationship between IBS-SSS and IBS-QoL, Spearman’s rank correlation coefficient was used to assess the direction and strength of the monotonic association between the total IBS-SSS and IBS-QoL scores. Statistical significance was determined at *p* < 0.05.

## 3. Result

The study enrolled 123 IBS-D patients with a mean age of 42.6 ± 14.5 years, of which 68 (55.3%) were females. Table 1 presents a detailed description of the characteristics of the study population. Regarding sociodemographic characteristics, the majority of patients were blue-collar workers (55.3%) and resided in urban areas (52.8%). The mean body mass index (BMI) of the study population was 23.1 ± 3.6 kg/m^2^.

Abdominal pain was the most common presenting complaint, reported by 60.2% of patients, followed by diarrhea at 33.3%. The median bowel movement frequency was 2 times per day. Regarding medical history and lifestyle habits, 32.5% of patients reported alcohol consumption and 15.5% reported smoking. Comorbid chronic conditions were relatively infrequent, with hypertension present in 13.1% and diabetes in 6.6% of the study population.

The prevalence of overlap syndromes among patients with IBS was notably high. The most common was overlap with functional dyspepsia (FD), observed in 46.3% of cases. Overlap with GERD was present in 8.9%, and 12.2% of patients were diagnosed with all three conditions concurrently (IBS + GERD + FD). Additionally, 24.4% of patients were found to have *H. pylori* infection.

The median IBS-SSS was 175 (interquartile range: 140–225). When classified by severity, the majority of patients fell into the moderate (46.3%) and mild (42.3%) categories, while 7.3% were categorized as having severe symptoms. The median quality of life score (IBS-QoL) in the study population was 72 (interquartile range: 54–85).

The analysis showed no statistically significant differences in age, gender, place of residence, occupation, or BMI between patients with low (<175) and high (≥175) IBS-SSS scores. Similarly, no significant differences were observed between patients with low (<72) and high (≥72) IBS-QoL scores. Comorbid factors such as hypertension, diabetes, smoking, and alcohol consumption were also evenly distributed across the comparison groups (*p* > 0.05).

The group with higher IBS-SSS scores had a significantly greater mean number of daily bowel movements (2.4 ± 1.3 times/day) compared to the group with lower IBS-SSS scores (1.8 ± 1.3 times/day) (*p* = 0.008). A statistically significant difference was also observed in the median scores for abdominal pain severity (*p* < 0.001) and bloating severity (*p* = 0.006) between the two groups. The study found a statistically significant difference in abdominal pain severity between the groups with low and high IBS-QoL scores (*p* = 0.012). However, no significant differences were observed between the two QoL groups in terms of bowel movement frequency, bloating severity, or chief complaints (abdominal pain vs. diarrhea) (*p* > 0.05). The prevalence of overlap syndromes (GERD, functional dyspepsia), *H. pylori* infection, and neuropsychological disorders (anxiety, insomnia) did not differ significantly when stratified by IBS-SSS severity levels or IBS-QoL scores (Table 2).

When patients were classified into severe (IBS-SSS ≥ 300) and non-severe (IBS-SSS < 300) groups, no statistically significant differences were observed in age, gender, place of residence, occupation, or BMI between the two groups (*p* > 0.05). However, in terms of medical history and lifestyle habits, the severe IBS group had a significantly higher smoking rate compared to the non-severe group (44.4% vs. 13.2%; *p* = 0.012). Additionally, the prevalence of diabetes was significantly higher in the severe IBS group (22.2% vs. 5.3%; *p* = 0.047). No significant differences were found between the severe and non-severe IBS groups in terms of chief complaints (abdominal pain or diarrhea), average bowel movement frequency, or bloating severity. However, a statistically significant difference in abdominal pain severity was observed between the two groups (*p* < 0.001). Patients in the severe group had poorer quality of life compared to those in the non-severe group, with median IBS-QoL scores of 52 vs. 74, respectively (*p* = 0.011) (Table 3).

**Figure 2:**
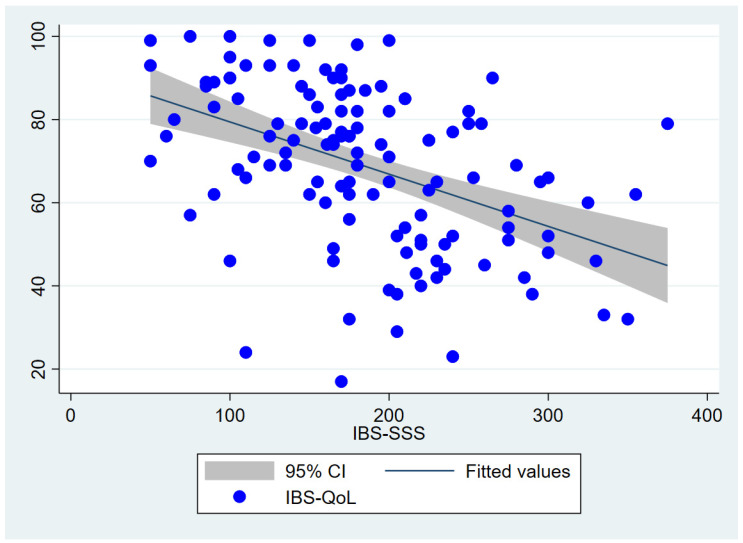
Correlation between IBS Symptom Severity Score and IBS Quality of Life. Abbreviations: CI, confidence interval; IBS-SSS, IBS Symptom Severity Score; IBS-QoL, IBS Quality of Life.

Spearman correlation analysis showed a moderate negative correlation between IBS-SSS and IBS-QoL scores, with r = −0.494 ((−0.638)–(−0.349), *p* < 0.001) (Figure 2).

## 4. Discussion

Our study of 123 Vietnamese patients with IBS-D provides real-world data on the symptom burden and impact of the disease on quality of life. The findings demonstrate that IBS-D is not merely limited to lower gastrointestinal symptoms but is also associated with a high prevalence of overlap syndromes. Moreover, disease severity, as measured by the IBS-SSS, showed a moderate inverse correlation with quality of life, as assessed by the IBS-QoL questionnaire.

In terms of demographic characteristics, the study recorded a female predominance (55.3%) with a mean age of 42.6 years. These findings are consistent with previous epidemiological data in Vietnam, which indicate that IBS is more common in women and typically occurs in individuals of working age (20–40 years) [9,11]. This pattern also aligns with global and regional trends reported in the Seoul Consensus 2025 and publications by the ROME Foundation, reaffirming that IBS is a prevalent disorder among women and younger adults [3,4].

A notable finding in this study is the high prevalence of overlap syndromes, particularly functional dyspepsia (FD), which affected 46.3% of patients, and gastroesophageal reflux disease (GERD), present in 8.9%. This high rate of overlap supports the modern view of IBS as a complex brain–gut interaction disorder rather than a condition confined solely to the colon [1,3]. A review by Quach et al. also reported that Vietnamese patients with IBS have nearly a threefold increased risk of comorbid FD and GERD compared to individuals without IBS [9]. The presence of these comorbid conditions, along with psychological disturbances such as anxiety and insomnia (as observed in our study), adds complexity to the clinical picture of IBS-D. This finding supports the conclusions of Lackner et al., who emphasized that the type of comorbidities—both physical and psychological—plays a more critical role in increasing symptom burden than the number of comorbid conditions alone [7].

Regarding disease severity, the median IBS-SSS score in our study was 175, corresponding to a moderate level of severity. Notably, we identified a moderate negative correlation between IBS-SSS and IBS-QoL scores (r = −0.494; *p* < 0.001). This indicates that the more severe the symptoms—such as frequent abdominal pain, bloating, and increased bowel movements—the greater the impairment in quality of life. Importantly, this relationship reflects the multidimensional nature of IBS, in which symptom severity affects not only physical discomfort but also psychological well-being, social functioning, and dietary behavior. The IBS-QoL instrument captures these domains, reinforcing its role as a comprehensive tool for assessing disease burden beyond gastrointestinal symptoms alone. These findings reaffirm the validity of the IBS-QoL scale in assessing the impact of IBS, consistent with the initial validation studies conducted by Drossman et al. [13]. The IBS-QoL questionnaire, with its multidomain structure, allows for a nuanced assessment of both physical and psychological impacts of IBS. In our cohort, the consistent inverse correlation with IBS-SSS further supports its applicability in the Vietnamese population and highlights its value in both clinical assessment and research settings. Moreover, the study by Trindade et al. also demonstrated that IBS severity negatively affects both physical and mental health, with gastrointestinal-specific anxiety playing a key mediating role in this relationship [14]. In comparison with a clinical trial conducted in Vietnam by Vo et al., the average pre-intervention quality of life score among IBS patients ranged from approximately 66 to 70, which is fairly consistent with the median score of 72 observed in our study [10]. This similarity underscores the consistent burden of IBS on quality of life across different Vietnamese patient populations.

A more in-depth analysis of factors associated with disease severity revealed that patients in the severe IBS group (IBS-SSS ≥ 300) had significantly higher rates of smoking and diabetes compared to the non-severe group. Although current guidelines, such as those from the British Society of Gastroenterology, recommend lifestyle modification as a first-line approach [1], the specific relationship between smoking and IBS symptom severity remains underexplored in clinical practice guidelines. However, smoking has been shown to exacerbate IBS symptoms through multiple mechanisms, including modulation of autonomic nervous system activity, increased intestinal permeability, and low-grade mucosal inflammation [15]. Nicotine may also influence gut motility and visceral sensitivity, thereby contributing to symptom amplification. These findings suggest that smoking may represent a modifiable risk factor associated with more severe IBS-D and should be considered in clinical management strategies.

To summarize, our study confirms that IBS-D significantly impairs the quality of life among Vietnamese patients, particularly those with severe symptoms and multiple comorbidities. The combined use of the IBS-SSS and IBS-QoL scales in clinical practice is essential for effective patient stratification and for optimizing treatment in accordance with the biopsychosocial model recommended by international consensus guidelines [1,2,4].

## 5. Limitations

This study has several limitations. First, its cross-sectional design allows for the identification of associations between disease severity and quality of life but does not establish causality. Second, the study was conducted at a tertiary referral center, which may introduce selection bias by overrepresenting patients with more severe disease compared to the general population. Third, the sample size was relatively modest (n = 123), with a particularly small number of patients in the severe group (n = 9), which may have limited the statistical power of subgroup analyses. Lastly, the assessment of psychological comorbidities relied on clinical diagnoses and self-reported questionnaires, which may be subject to recall bias or underestimation of the true prevalence. Additionally, the lack of detailed treatment-related data may have influenced both symptom severity and quality-of-life outcomes.

## 6. Conclusions

This study confirms that IBS-D has a substantial impact on the quality of life of patients in Vietnam. The findings demonstrate a moderate inverse correlation between symptom severity (IBS-SSS) and quality of life (IBS-QoL), while also highlighting a high prevalence of overlap syndromes-particularly functional dyspepsia and gastroesophageal reflux disease. Notably, smoking and diabetes were identified as factors closely associated with patients experiencing more severe symptoms.

Therefore, the routine use of IBS-SSS and IBS-QoL scoring systems in clinical practice is essential for accurate patient stratification. Management strategies for IBS-D should shift toward a comprehensive, biopsychosocial approach that not only targets gastrointestinal symptom control but also addresses comorbidities and lifestyle interventions to optimize long-term treatment outcomes.

## Figures and Tables

**Table 1:** Characteristics of the study population.

Characteristics	Total (n = 123)
**Age (years)**, mean (±SD)	42.6 ± 14.5
**Female**, n (%)	68 (55.3)
**Residential area**, n (%)	
Urban (city/town)	65 (52.8)
Rural	58 (47.2)
**Occupation**, n (%)	
Blue-collar	68 (55.3)
White-collar	55 (44.7)
**History**, n (%)	
Hypertension	16 (13.1)
Diabetes	8 (6.6)
Smoking	19 (15.5)
Using alcohol	40 (32.5)
**BMI**, mean (±SD)	23.1 ± 3.6
**Chief complaint**, n (%)	
Abdominal pain	74 (60.2)
Diarrhea	41 (33.3)
Other	8 (6.5)
**Stool frequency/day**, median	2 (1–3)
**Overlap syndrome**, n (%)	
GERD	11 (8.9)
Functional dyspepsia (FD)	57 (46.3)
GERD + FD	15 (12.2)
***H. pylori* infection**, n (%)	30 (24.4)
**IBS-SSS**, median (interquartile range)	175 (140–225)
<75 (remission), n (%)	5 (4.1)
75–174 (mild), n (%)	52 (42.3)
175–299 (moderate), n (%)	57 (46.3)
≥300 (severe), n (%)	9 (7.3)
**IBS-QoL**, median (interquartile range)	72 (54–85)

BMI, body mass index; FD, functional dyspepsia; GERD, gastroesophageal reflux disease; IBS-SSS, IBS symptom severity score; IBS-QoL, IBS quality of life; SD, standard deviation.

**Table 2:** Comparison of clinical and demographic characteristics by IBS Severity (IBS-SSS) and Quality of Life (IBS-QoL).

Characteristics	Total (n = 123)	Low IBS-SSS (<175) (n = 57)	High IBS-SSS (≥175) (n = 66)	*p*	Low IBS-QoL (<72) (n = 61)	High IBS-QoL (≥72) (n = 62)	*p*
**Age (years)**, mean (±SD)	42.6 ± 14.5	43.7 ± 13.3	41.7 ± 15.6	0.459	43.1 ± 15.9	42.1 ± 13.1	0.699
**Female**, n (%)	68 (55.3)	31 (54.4)	37 (56.1)	0.852	32 (52.5)	36 (58.1)	0.532
**Residential area**, n (%)							
Urban (city/town)	65 (52.8)	31 (54.4)	34 (51.5)	0.75	25 (41.0)	33 (53.2)	0.174
Rural	58 (47.2)	26 (45.6)	32 (48.5)	0.75	36 (59.0)	29 (46.8)	0.174
**Occupation**, n (%)							
Blue-collar	68 (55.3)	32 (56.1)	36 (54.6)	0.859	31 (50.8)	37 (59.7)	0.323
White-collar	55 (44.7)	25 (43.9)	30 (45.6)	0.859	30 (49.2)	25 (40.3)	0.323
**History**, n (%)							
Hypertension	16 (13.1)	4 (7.02)	12 (18.2)	0.066	7 (11.5)	9 (14.5)	0.616
Diabetes	8 (6.6)	4 (7.02)	4 (6.06)	0.83	5 (8.2)	3 (4.8)	0.45
Smoking	19 (15.5)	9 (15.8)	10 (15.2)	0.922	10 (16.4)	9 (14.5)	0.773
Using alcohol	40 (32.5)	17 (29.8)	23 (34.85)	0.553	17 (27.9)	23 (37.1)	0.275
**BMI**, mean (±SD)	23.1 ± 3.6	22.8 ± 3.8	23.3 ± 3.4	0.507	23.1 ± 3.9	23.0 ± 3.4	0.963
**Chief complaint**, n (%)							
Abdominal pain	74 (60.2)	31 (54.4)	43 (65.2)	0.224	36 (59.0)	38 (61.3)	0.797
Diarrhea	41 (33.3)	23 (40.4)	18 (27.3)	0.125	20 (32.8)	21 (33.9)	0.899
Other	8 (6.5)	3 (5.3)	5 (7.6)	0.604	5 (8.2)	3 (4.8)	0.45
**Stool frequency/day**, mean (±SD)	2.1 ± 1.4	1.8 ± 1.3	2.4 ± 1.3	**0.008**	2.3 ± 1.5	2.0 ± 1.2	0.215
**Abdominal pain level**, median	3 (2–4)	4 (3–4)	3 (2–3)	**<0.001**	3 (2–4)	3 (3–4)	**0.012**
**Bloating level**, median	3 (3–4)	4 (3–4)	3 (3–4)	**0.006**	3 (3–4)	3 (3–4)	0.122
**Overlap syndrome**, n (%)							
GERD	11 (8.9)	6 (10.5)	5 (7.6)	0.567	6 (9.8)	5 (8.1)	0.731
Functional dyspepsia	57 (46.3)	25 (43.9)	32 (48.5)	0.608	27 (44.3)	30 (48.4)	0.646
GERD + FD	15 (12.2)	9 (15.8)	6 (9.1)	0.258	5 (8.2)	10 (16.1)	0.179
***H. pylori* infection**, n (%)	30 (24.4)	16 (28.1)	14 (21.2)	0.377	13 (21.3)	17 (27.40	0.43
**Neuropsychiatric disorders**, n (%)							
Anxiety disorder	5 (4.1)	3 (5.3)	2 (3.0)	0.532	1 (1.6)	4 (6.5)	0.177
Insomnia	3 (2.4)	2 (3.5)	1 (1.5)	0.475	2 (3.3)	1 (1.6)	0.549

BMI, body mass index; FD, functional dyspepsia; GERD, gastroesophageal reflux disease; IBS-SSS, IBS symptom severity score; IBS-QoL, IBS quality of life; SD, standard deviation. Bold values indicate statistically significant differences (*p* < 0.05), based on the corresponding statistical tests.

**Table 3:** Comparison of clinical characteristics and quality of life between severe and non-severe IBS groups.

Characteristics	Total (n = 123)	Non-Severe IBS-SSS < 300 (n = 114)	Severe IBS-SSS ≥ 300 (n = 9)	*p*
**Age (years)**, mean (±SD)	42.6 ± 14.5	42.7 ± 14.3	41.1 ± 18.1	0.644
**Female**, n (%)	68 (55.3)	63 (55.3)	5 (55.6)	0.986
**Residential area**, n (%)				
Urban (city/town)	65 (52.8)	60 (52.6)	5 (55.6)	0.866
Rural	58 (47.2)	54 (47.4)	4 (44.4)	0.866
**Occupation**, n (%)				
Blue-collar	68 (55.3)	63 (55.3)	5 (55.6)	0.986
White-collar	55 (44.7)	51 (44.7)	4 (44.4)	0.986
**History**, n (%)				
Diabetes	8 (6.6)	6 (5.3)	2 (22.2)	**0.047**
Smoking	19 (15.5)	15 (13.2)	4 (44.4)	**0.012**
Using alcohol	40 (32.5)	37 (32.5)	3 (33.3)	0.957
**BMI**, mean (±SD)	23.1 ± 3.6	23.2 ± 3.7	21.1 ± 1.9	0.076
**Chief complaint**, n (%)				
Abdominal pain	74 (60.2)	67 (58.8)	7 (77.8)	0.262
Diarrhea	41 (33.3)	40 (35.1)	1 (11.1)	0.142
Other	8 (6.5)	7 (6.1)	1 (11.1)	0.56
**Stool frequency/day**, mean (±SD)	2.1 ± 1.4	2.1 ± 1.4	2.2 ± 1.3	0.893
**Abdominal pain level**, median	3 (2–4)	3 (3–4)	1 (1–2)	**<0.001**
**Bloating level**, median	3 (3–4)	3 (3–4)	3 (2–4)	0.105
**Overlap syndrome**, n (%)				
GERD	11 (8.9)	9 (7.9)	2 (22.2)	0.147
Functional dyspepsia	57 (46.3)	53 (46.5)	4 (44.4)	0.906
GERD + FD	15 (12.2)	15 (13.2)	0 (0.0)	0.246
**IBS-QoL**, median	72 (54–85)	74 (57–85)	52 (46–62)	**0.011**

BMI, body mass index; FD, functional dyspepsia; GERD, gastroesophageal reflux disease; IBS-SSS, IBS symptom severity score; IBS-QoL, IBS quality of life; SD, standard deviation.

## Data Availability

The datasets used and/or analyzed during the current study are available from the corresponding author on reasonable request.

## Abbreviations

BMI, body mass index; FD, functional dyspepsia; GERD, gastroesophageal reflux disease; IBS, irritable bowel syndrome; IBS-D, diarrhea-predominant irritable bowel syndrome; IBS-SSS, IBS symptom severity score; IBS-QoL, IBS quality of life; QoL, quality of life; SD, standard deviation.
